# Garlic Supplementation Ameliorates UV-Induced Photoaging in Hairless Mice by Regulating Antioxidative Activity and MMPs Expression

**DOI:** 10.3390/molecules21010070

**Published:** 2016-01-08

**Authors:** Hye Kyung Kim

**Affiliations:** Department of Food & Biotechnology, Hanseo University, Haemi-Myun, Seosan, Chungnam 356-706, Korea; hkkim111@dreamwiz.com; Tel.: +82-41-660-1454

**Keywords:** photoaging, UV irradiation, garlic, MMPs, type 1 procollagen

## Abstract

UV exposure is associated with oxidative stress and is the primary factor in skin photoaging. UV-induced reactive oxygen species (ROS) cause the up-regulation of metalloproteinase (MMPs) and the degradation of dermal collagen and elastic fibers. Garlic and its components have been reported to exert antioxidative effects. The present study investigated the protective effect of garlic on UV-induced photoaging and MMPs regulation in hairless mice. Garlic was supplemented in the diet, and Skh-1 hairless mice were exposed to UV irradiation five days/week for eight weeks. Mice were divided into four groups; Non-UV, UV-irradiated control, UV+1% garlic powder diet group, and UV+2% garlic powder diet group. Chronic UV irradiation induced rough wrinkling of the skin with hyperkeratosis, and administration of garlic diminished the coarse wrinkle formation. UV-induced dorsal skin and epidermal thickness were also ameliorated by garlic supplementation. ROS generation, skin and serum malondialdehyde levels were significantly increased by UV exposure and were ameliorated by garlic administration although the effects were not dose-dependent. Antioxidant enzymes such as superoxide dismutase and catalase activities in skin tissues were markedly reduced by UV irradiation and garlic treatment increased these enzyme activities. UV-induced MMP-1 and MMP-2 protein levels were suppressed by garlic administration. Furthermore, garlic supplementation prevented the UV-induced increase of MMP-1 mRNA expression and the UV-induced decrease of procollagen mRNA expression. These results suggest that garlic may be effective for preventing skin photoaging accelerated by UV irradiation through the antioxidative system and MMP regulation.

## 1. Introduction

Chronic exposure to sunlight is known to induce the premature aging of the skin (*i.e.*, photoaging) characterized by wrinkles, roughness, mottled pigmentation and histological changes that include increased epidermal thickness and connective tissue alteration [1]. Wrinkle formation is a striking feature of photoaged skin and is caused by the degradation and degeneration of collagen and the accumulation of altered elastic fibers in the dermis [2].

Excessive exposure to UV irradiation generates reactive oxygen species (ROS) in the skin [3], and overproduction of ROS can overwhelm the antioxidant-defense mechanism, resulting in oxidative stress and oxidative photo-damage in the skin [4]. An enzymatic antioxidant defense system composed of catalase and superoxide dismutase (SOD) is therefore crucial for the protection of the skin from UV-induced oxidative stress [5]. Recently, it was demonstrated in human skin that acute and chronic photo-damage is accompanied by depleted antioxidant enzyme expression and increased oxidative protein modifications [6]. Photoaging is also characterized by the accumulation of lipid peroxidation and glycation products [7].

ROS lead to the activation of transcription factors which causes up-regulation of metalloproteinase (MMPs) and degradation of dermal collagen and elastic fibers [8]. MMP-1 and MMP-2, also known as type I and IV collagenase, respectively, are up-regulated and serve as the primary MMPs in UV-exposed skin. Therefore, the excessive degradation of collagen and matrix by UV-induced MMPs is a characteristic feature of photo-damaged skin, and MMP is used for a major marker of UV-induced photoaging as well as skin inflammation [9].

Garlic has long been used widely not only as a flavoring agent but also as a folk medication and is one of the most well-known herbal medicines worldwide. Numerous therapeutic effects of garlic and its constituents such as antioxidant, anti-microbial, anti-atherosclerotic, anti-diabetic, anti-mutagenic, anti-carcinogenic and immune-modulation activities have been reported [10,11,12,13,14,15]. However, the effect on photoaging has not yet been elucidated. Therefore, the present study examined the protective effects of garlic on UV-induced skin aging in a hairless mouse model, and elucidated the underlying mechanisms responsible for such an effect.

## 2. Results

### 2.1. Body Weight and Serum Biochemical Markers

The changes of body weight and serum biochemical indicators in the hairless mice were determined to examine the animal toxicity of garlic powder. Mean body weights of all experimental groups were similarly increased throughout the period of study. There were no significant differences in body weight and food intake between the groups during the eight weeks of the experimental periods (data not shown), suggesting that body weight gain and food consumption were not affected by UV irradiation and garlic supplementation.

**Figure 1:**
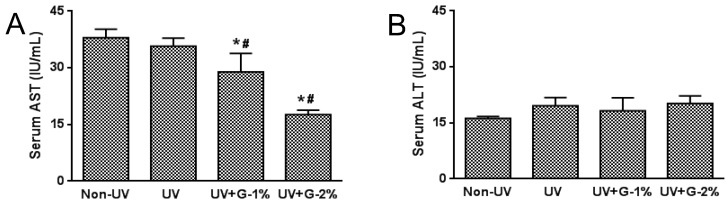
Effects of garlic on serum biochemical parameters for hepatotoxicity in UV-exposed hairless mice. (**A**) Serum aspartate transaminase (AST) and (**B**) alanine transaminase (ALT) activity in UV-exposed hairless mice. Each bar represents the mean ± SD (*n* = 8). Non-UV, normal group; UV, UV-irradiated control group; UV+G-1%, UV-irradiated and 1% (*w*/*w*) dietary supplementation group; UV+G-2%, UV-irradiated and 2% (*w*/*w*) dietary supplementation group. * *p* < 0.05 *vs.* Non-UV group; # *p* < 0.05 *vs.* UV control group.

The hepatotoxicity of garlic was determined by measuring the serum aspartate transaminase (AST) and alanine transaminase(ALT) concentrations (Figure 1). UV irradiation fails to affect serum AST and ALT levels. However, AST concentrations were dose-dependently decreased by garlic supplementation while ALT concentrations were not affected by garlic treatment.

### 2.2. Wrinkle Formation and Skin Thickening

**Figure 2:**
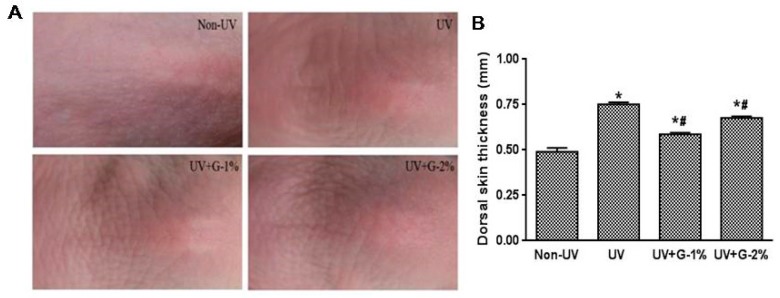
Effects of garlic on wrinkle formation and skin thickness of UV-exposed hairless mice. (**A**) Representative images of macroscopic morphological observation on skin of hairless mice. The dorsal skin surfaces of hairless mice were mock-irradiated or UV-irradiated five days/week for eight weeks; (**B**) Thickness of dorsal skin was measured using a caliper after eigh weeks. Each bar represents the mean ± SD (*n* = 8). Non-UV, normal group; UV, UV-irradiated control group; UV+G-1%, UV-irradiated and 1% (*w*/*w*) dietary supplementation group; UV+G-2%, UV-irradiated and 2% (*w*/*w*) dietary supplementation group. * *p* < 0.05 *vs.* Non-UV group; # *p* < 0.05 *vs.* UV control group.

The effect of garlic on UV-induced wrinkle formation was observed by visual assessment of close-up photos in the dorsal skins of hairless mice. Chronic UV irradiation induced deep, irregular wrinkling of the skin with hyperkeratosis, and marked reduction in UV-induced wrinkle formation was observed in the UV+G-1% and UV+G-2% groups compared with the UV group (Figure 2A).

Chronic UV irradiation induces skin inflammation, which causes excessive epidermal proliferation and skin thickening [16]. The effect of UV irradiation on skin thickening was examined by measuring the skin-fold thickness of the dorsal skin using a caliper. As shown in Figure 2B, UV irradiation significantly increased (1.5-fold) the skin-fold thickness. However, administration of garlic resulted in significant reduction in dorsal skin thickness. G-1% and G-2% groups reduced the skin thickness by 32.1% ± 1.3% and 10.2% ± 1.2%, respectively, compared with the UV control group. The result suggests that 1% dietary supplementation of garlic was more effective than 2% supplementation.

### 2.3. Histopathological Changes

**Figure 3:**
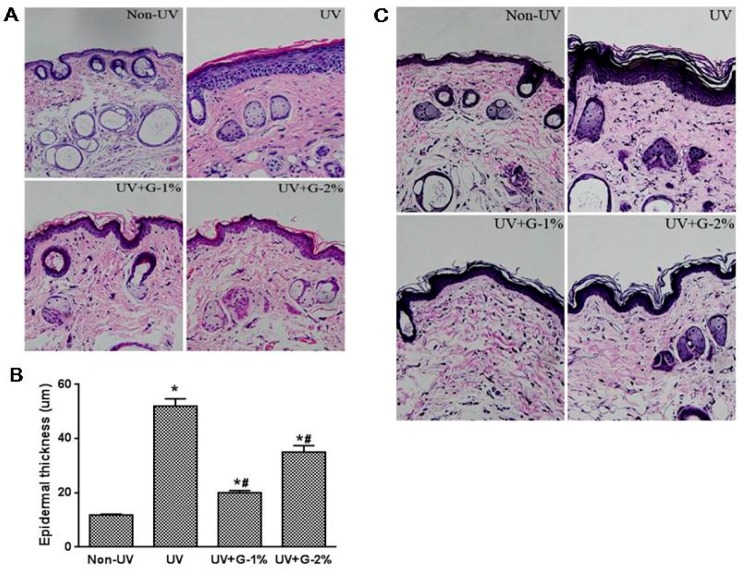
Histological analysis of dorsal skin in garlic-treated mice against UV-induced skin damage. (**A**) Photomicrographs of H & E-stained skin sections, representing epidermal thickness; (**B**) Epidermal thickness was measured from the H&E-stained sections; (**C**) Verhoeff Van Gieson staining. Original magnification ×400. Each bar represents the mean ± SD (*n* = 8). Non-UV, normal group; UV, UV-irradiated control group; UV+G-1%, UV-irradiated and 1% (*w*/*w*) dietary supplementation group; UV+G-2%, UV-irradiated and 2% (*w*/*w*) dietary supplementation group. * *p* < 0.05 *vs.* Non-UV group; # *p* < 0.05 *vs.* UV control group.

Sections of dorsal skin from hairless mice were stained with Hematoxylin and eosin (H & E) or the Verhoeff van-Gieson method to determine epidermal hyperplasia and elastic fiber, respectively. Epidermal thickness, as a histological feature of photoaged skin, can be used as one of the quantitative parameters to reflect UV-induced skin damage, since thickened epidermis can contribute to skin roughness [17]. Based on the H & E stain of dorsal skin, the thickness of the epidermis (stained in dark purple) was observed (Figure 3A). Compared with normal group, the UV-irradiated group showed a marked increase in epidermal thickness due to hyperplasia/hypertrophy of the epidermal keratinocytes. Furthermore, infiltration of the inflammatory cells was found in the dermis of UV-exposed skin tissue. Dietary supplementation of garlic (1% and 2%) ameliorated the UV-induced epidermal thickness and epidermal hypertrophy as well as the inflammatory cells in the dermis (Figure 3A). In a quantitative analysis, histological measurements of the epidermal thickness demonstrated that UV irradiation induced a 4.4-fold increase in the epidermal thickness of dorsal skin compared with the non-irradiated group (Figure 3B). Garlic treatment (1%) decreased the UV-induced epidermal thickening by 61.4% ± 1.5% while G-2% reduced the epidermal thickness by 32.5% ± 4.5% (*p* < 0.05). The results again showed that 1% garlic supplementation exhibited better protection from UV-induced skin damage.

Elastic fibers are important components of the skin and are responsible for skin elasticity. To visualize the changes in elastic fibers in the dermal areas, histological sections of the skin were subjected to Verhoeff’s staining. As shown in Figure 3C, fine elastic fibers (stained in blue-black to black) perpendicular and parallel to the body axis were observed in the lower dermis in the normal non-irradiated group. Elastic fibers were elongated and slender with a homogeneous distribution. On the other hand, in UV-irradiated skin, elastic fibers in the lower dermis were highly accumulated and appeared short, thickened and twisted. However, compared to the UV-irradiated control group, the tortuosity of elastic fibers showed a significant difference in the 1% garlic-administered group. UV irradiation caused the elastic fibers to break in the dermis and garlic could protect the elastic fibers in UV-exposed mice skin. In addition to elastic fiber degeneration, reduction in the dermis collagen fiber density (stained in pink) as well as abnormal collagen deposition was observed by UV exposure. Garlic treatment, especially G-1%, significantly improved collagen fiber density (Figure 3C). Collagen fibers in the normal group displayed a highly dense regular arrangement while they were severely broken and reduced in the structural density in the dermis of the UV-irradiated group. As expected, collagen fibers in the dermis of the G-1% group were almost intact with a regular arrangement (Figure 3C). Therefore, garlic can protect both the epidermis and the dermis of skin exposed to chronic UV irradiation.

### 2.4. Antioxidant Status

**Figure 4:**
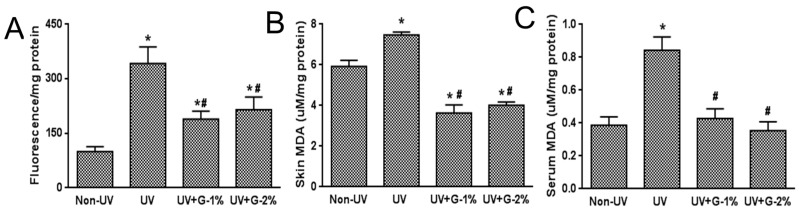
Effects of garlic on ROS and lipid peroxidation in UV-exposed hairless mice. (**A**) Total ROS level was determined using DCFDA probe in hairless mice skin homogenates. The levels of malondialdehyde (MDA) in the skin (**B**) and serum (**C**) were estimated by the thiobarbituric acid assay. Each bar represents the mean ± SD (*n* = 8). Non-UV, normal group; UV, UV-irradiated control group; UV+G-1%, UV-irradiated and 1% (*w*/*w*) dietary supplementation group; UV+G-2%, UV-irradiated and 2% (*w*/*w*) dietary supplementation group. * *p* < 0.05 *vs.* Non-UV group; # *p* < 0.05 *vs.* UV control group.

Oxidative stress is a primary factor in the photoaging process. UV increases levels of hydrogen peroxides and other reactive oxygen species in skin and decreases the levels of antioxidant enzymes [3,6]. Increased ROS production alters gene and protein structures and functions, and ultimately leads to skin damage. To assess UV-induced oxidative statuses, total ROS level, lipid peroxidation, and antioxidant defense enzymes such as SOD and catalase were determined. ROS generation in skin tissue was markedly increased by UV exposure and the UV-induced ROS level was suppressed by garlic administration (Figure 4A). However, a dose-dependent effect of garlic was not observed.

Malondialdehyde (MDA) is the aldehydic decomposition product of lipid oxidation, which is the major thiobarbituric acid reactive substance (TBARS). The lipid peroxide levels in skin and serum were determined by measuring the TBARS concentration. As shown in Figure 4B,C, MDA levels in the skin and serum of UV-irradiated mice were significantly increased by 26% and 218%, respectively, compared to the normal control group. The increased skin and serum MDA levels caused by UV irradiation were suppressed by 49.3% and 51.5%, respectively, in the 1% garlic-supplemented group. The results showed that the effect of UV was greater in the serum than the skin and the effect of garlic was not dose-dependent.

**Figure 5:**
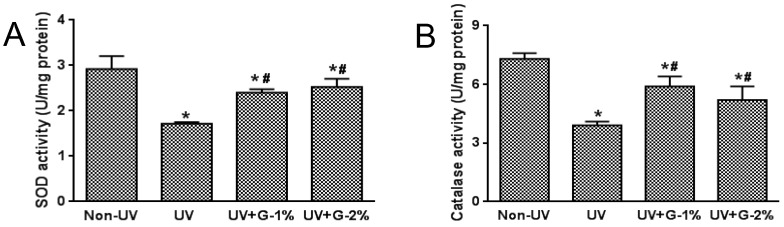
Effects of garlic on antioxidant enzyme activities in UV-exposed hairless mice. (**A**) Superoxide dismutase (SOD) activity and (**B**) catalase activity was expressed as U/mg protein. Each bar represents the mean ± SD (*n* = 8). Non-UV, normal group; UV, UV-irradiated control group; UV+G-1%, UV-irradiated and 1% (*w*/*w*) dietary supplementation group; UV+G-2%, UV-irradiated and 2% (*w*/*w*) dietary supplementation group. * *p* < 0.05 *vs.* Non-UV group; # *p* < 0.05 *vs.* UV control group.

Serum SOD and catalase activities were significantly reduced by UV irradiation, while dietary garlic supplementation significantly increased these enzyme activities compared with the UV-irradiated control group (Figure 5). No significant difference was observed among the G-1% and G-2% groups. SOD and catalase activities in skin tissue showed a similar trend as the serum (data not shown). These results indicated that garlic was able to reduce oxidative stress in the skin as well as serum through improvement of the ROS level, lipid peroxidation, and antioxidant enzyme activities.

### 2.5. MMP Protein Expression

**Figure 6:**
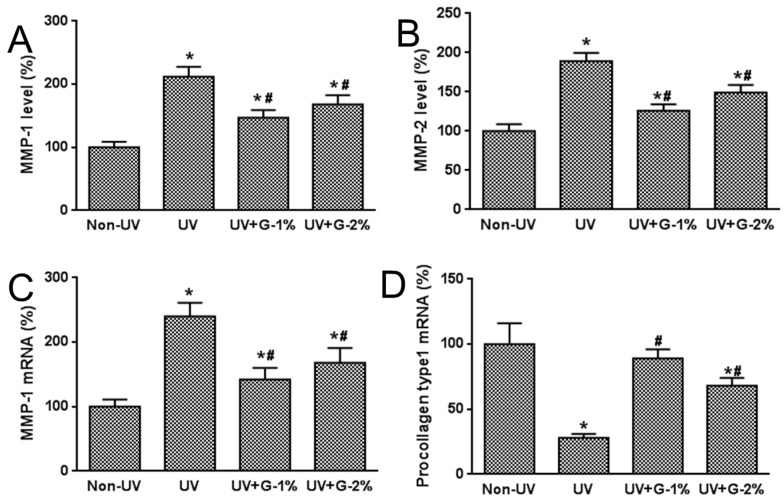
Effects of garlic on MMPs and procollagen type 1 expressions in UV-exposed hairless mice. (**A**) MMP-1 and (**B**) MMP-2 productions in skin homogenates were determined by ELISA. MMP-1 (**C**) and procollagen type 1 (**D**) mRNA expressions were determined by quantitative real-time RT-PCR. Data are expressed as % of Non-UV normal group. Each bar represents the mean ± SD (*n* = 8). Non-UV, normal group; UV, UV-irradiated control group; UV+G-1%, UV-irradiated and 1% (*w*/*w*) dietary supplementation group; UV+G-2%, UV-irradiated and 2% (*w*/*w*) dietary supplementation group. * *p* < 0.05 *vs.* Non-UV group; # *p* < 0.05 *vs.* UV control group.

MMPs (especially MMP-1 and MMP-2) have been known to play important roles in the degradation of skin collagen and elastin. To investigate the mechanism underlying the protective effect of garlic on UV-induced photoaging, expressions of MMP-1, MMP-2, and procollagen type 1 were analyzed (Figure 6). The results demonstrated that UV irradiation induced a marked increase in protein levels of MMP-1 and MMP-2,as assessed by ELISA analysis, by 212% and 89%, respectively. Dietary supplementation of garlic caused a significant decrease in MMP-1 and MMP-2 levels, but no significant differences were observed between the G-1% and G-2% groups.

Type 1 collagen is the most abundant collagen in skin connective tissue as well as the human body, and newly synthesized procollagen type 1 is secreted into the dermal extracellular space, and has an anti-wrinkle effect in UV-irradiated skin. Also, a decrease in procollagen expression caused by repeated UV irradiation has been considered to be a cause of photoaging [18]. Therefore, the effects of garlic on mRNA expression of MMP-1 and type 1 procollagen were investigated using quantitative real-time RT-PCR analysis. MMP-1 mRNA expression was dramatically induced while procollagen mRNA expression was decreased by UV exposure. Garlic supplementation prevented the UV-induced increase of MMP-1 expression and the UV-induced decrease of procollagen mRNA expression. These results indicate that the decreased expression of procollagen type 1 due to UV exposure was caused by the over-expression of UV-induced MMP-1.

## 3. Discussion

One of the most important biochemical properties of garlic is its antioxidant potential. Garlic or garlic oil supplementation prevented the increase in oxidative stress associated with gentamicin-induced nephrotoxicity or nicotine-induced lipid peroxidation in rats [19,20]. Oxidative stress was ameliorated by preserving SOD, catalase, and glutathione peroxidase activities. Lau [13] demonstrated that some components of garlic, *S*-allylcysteine and allixin, suppress LDL oxidation *in vitro*. Furthermore, short-term supplementation of garlic in human subjects has demonstrated an increased resistance of LDL to oxidation as well as blood antioxidant parameters [13,14].

Chronic exposure to UV irradiation results in photoaging of skin including various characteristic changes, such as DNA damage [2,21,22], histological changes [2,23], and antioxidant enzyme changes [3,5,6]. The major aspects of the pathogenesis of skin photoaging induced by UV include skin wrinkle formation, epidermal thickening, and degradation of MMPs [1,2,8]. Because these processes in UV-induced skin photoaging could be inhibited by antioxidants of natural origin [5,24], efforts have been made to identify antioxidants that could protect against photoaging due to UV exposure. The present study demonstrated the anti-photoaging properties of garlic in UV-exposed hairless mice.

Skin consists of three layers: the epidermis, the dermis, and the subcutis. The outer layer, the epidermis, is composed of keratinocytes, melanocytes, and langerhans cells. This layer serves as a barrier to protect the body by synthesis of the protein keratin. The dermis is made up of collagen fiber, elastin fiber, and the derma matrix, which are produced by fibroblasts [25]. The epidermis, exposed to UV irradiation, has an irregular shape, large follicles, excessive deposition of keratin, and an increase in epidermal thickness for protection of the dermis from UV damage. In the dermal areas, UV irradiation induces degeneration of collagen, extracellular matrix proteins, and elastic fibers, which lead to formation of wrinkles and loss of elasticity in skin [26]. Therefore, elastin accumulation and collagen degradation are prominent characteristics in photo-damaged skin. Some researchers demonstrated that decreases in skin elasticity, accompanied by the tortuosity of elastic fibers and loss of collagen fibers, were important early events in wrinkle formation [27,28]. In the present study, UV irradiation caused deep wrinkle formation and histopathological changes, characterized by epidermal hypertrophy, collagen fiber loss and an amorphous mass of abnormal elastic fibers. However, dietary supplementation of garlic attenuated macroscopic and histopathological changes induced by UV irradiation, suggesting that garlic supplementation in the diet has a photo-protective effect and anti-wrinkle activity.

Photo-oxidative damage caused by chronic UV exposure is the leading cause of extrinsic aging of the skin. In normal cells, antioxidative defense systems, including SOD, catalase, and glutathione peroxidase, have evolved to quench ROS [5,6,24]. However, when the ROS production level reaches above threshold, the overproduction of ROS can disturb the balance between ROS production and antioxidant defenses, which aggravate a decrease in activities of antioxidant enzymes. Several studies have reported that ROS overproduction can induce skin damage and photoaging during chronic UV irradiation [3,5,6,16,17]. Okada *et al.* [27] demonstrated that repeated exposure to UVB suppressed activity of SOD, which catalyzes the dismutation of superoxide into oxygen and hydrogen peroxide. Hydrogen peroxide, produced by SOD activity, is decomposed to water and oxygen by catalase activity. Low catalase activity has been observed in the skin of photoaging induced by UV [27]. In the present study, dietary supplementation of garlic exhibited protection against the UV-induced generation of ROS and lipid peroxidation, as well as reduction of antioxidant enzyme activities.

Photoaging is characterized by the degradation of collagen and the accumulation of abnormal elastin in the dermis, and several MMPs have been implicated in this process [8,9,29]. In particular, UV exposure is known to induce the expression of MMP-1, -2, -3, and -9 in normal human epidermis *in vivo* [9,29]. Also, UV exposure impairs collagen synthesis, primarily through the down-regulation of type 1 procollagen expression [29]. The present study showed that UV irradiation resulted in an increase in the protein level of MMP-1 and MMP-2 and a decrease in the mRNA expression of procollagen. A dietary supplement of garlic was found to cause a significant decrease in the protein and mRNA expression of MMP-1, as well as the protein level of MMP-2. Procollagen type 1 mRNA expression was increased by garlic treatment. The results of the present study indicate that garlic attenuates the UV-induced changes of MMPs and procollagen type-1 expressions, resulting in an improvement of collagen status and inhibition of wrinkle formation. In addition to collagen degradation, abnormal accumulation of elastic fibers and epidermal hyperplasia are also considered to be key histopathological changes associated with the photoaging effect of UV irradiation [30]. Collagen fibers are degraded by MMP-1 and MMP-2 and the elastin fibers by elastases, MMP-2, and MMP-9 [31]. Similarly, accumulation of elastic fibers in the dermis and an increase in epidermal thickness in the chronically UV-irradiated mice were observed, and garlic treatment led to a significant suppression of UV-induced accumulation of elastic fibers and epidermal hyperplasia (Figure 3C). Although the direct role of UV-induced MMPs in elastic fiber accumulation is still not clearly understood, it has been previously suggested that an increased number of elastic fibers synthesized in response to UV irradiation may replace the degraded collagen fibers in the dermis [29]. It is therefore possible that the suppression of collagen fiber degradation by garlic may prevent the accumulation of elastic fibers.

Garlic contains unique organosulfur compounds, which provide its characteristic flavor/odor and most of its potent biological activity. The main sulfur compound in both raw garlic and garlic powder is alliin, and allicin was found to be a major constituent of solvent extracted garlic [32]. Allicin is formed from alliin by the action of allinase and gets metabolized rapidly into diallyl sulfide, diallyl disulfide (DADS), dially ltrisulfide, ajoene, *S*-allylcysteine (SAC), and vinyl dithiines [32].

Antioxidative effects of sulfur compounds such as allicin, allyl disulfide, allyl cycteine, alliumthiosulfinates and *S*-alk(en)yl-l-cysteine sulfoxides have been reported [14,33,34]. Kim *et al.* reported that topical treatment of *S*-allyl cysteine, the most abundant component of aged garlic, exhibits antioxidative, anti-wrinkle activity, and down-regulates MMP-3, -9, and -12 protein expression [35]. However, a variety of compounds, including nonsulfur compounds, work synergistically to provide the health benefits of garlic. In fact, the majority of dried garlic is composed of fructose-containing carbohydrates (85%), followed by sulfur compounds, protein, fiber and free amino acids [36]. Recently, Chen *et al.* [37] reported the protective effect of fructan from white garlic against UV-induced cell damage in keratinocytes.

Bioavailability of active ingredients in garlic is likely essential, and it was suggested that the metabolites of allicin, rather than the allicin itself, are responsible for the health effects of garlic. SAC is one of the water-soluble organosulfur compounds in garlic. SAC absorbed rapidly and showed almost 100% bioavailability after oral administration [38]. Also, these metabolites have been reported to exhibit antioxidative activity. The oil-soluble organosulfur compounds in garlic, including allicin, sulfides, ajoene and vinyldithiins, are not found in blood or urine, even after consumption of a large amount (25 g of raw garlic; 90 mg allicin) of garlic [39]. Water-soluble SAC is a stable compound which lowers blood cholesterol [15], serve as an antioxidant [40,41], and inhibit the cancer process [42,43]. Other metabolites of garlic constituents, such as *N*-acetyl-*S*-(2-carboxypropyl)-cysteine, *N*-acetylcysteine and hexahydrohippuric acid, have been detected in human urine after ingestion of garlic [44]. At present, SAC is the only reliable human compliance marker used for studies involving garlic consumption because it is detectable and increases quantitatively in the blood after the oral intake of garlic capsules [45]. However, comparing the content of garlic products for their ability on the basis of certain compounds may be inadequate because other compounds may act synergistically or independently to bring about an effect.

The doses of garlic supplemented in the present study were 1% and 2% of the diet (*w*/*w*), and 1% garlic supplementation exhibited better or similar effect to 2% garlic supplementation, suggesting that 1% garlic supplementation reached the peak effective dose. The 1% garlic supplementation in the present study was 250 mg/kg/day garlic powder (food intake was about 5 g/day/mice) and was equivalent to approximately 4.5 g~5.7 g garlic powder/day for humans on the bases of energy requirements (energy requirement/day for human; 2000–2500 kcal/day, food intake; 22 kcal/day/mice). Pitana *et al.* [46] demonstrated that 250 mg/kg/day garlic extract supplementation exhibited greater effect than 500 mg/kg/day group on tissue MDA levels, plasma cholesterol level, and cognitive deficit in obese-insulin resistant rats.

## 4. Materials and Methods

### 4.1. Preparation of Garlic Powder

Fresh garlic (*Allium sativum* L.), purchased from Seosan (Chungnam, Korea) in July 2009, were peeled, vacuum dried, and powdered.

### 4.2. Experimental Animals and UV Irradiation

Six-week-old female albino hairless mice (Skh:HR-1) were obtained from Central Lab Animals, Inc. (Seoul, Korea). The animals were housed under controlled temperature (23 ± 2 °C), humidity (55% ± 5%) and light (12 h light/dark cycles). All animals had free access to water and commercial pellet food. After an acclimation period (one week), mice were randomly divided into four groups of eight animals: Non-UV, UV-irradiated control, UV+G-1% (UV+1% garlic powder diet group), UV+G-2% (UV+2% garlic powder diet group). Garlic powder was supplemented (1% or 2%, *w*/*w*) in commercial mouse diet (Samtako Korea, NIH#31M Diet). Body weight and food intake were regularly measured throughout the study. The animal protocol used in this study was reviewed and approved by the Kyunghee University Institutional Animal Care and Use Committee (KUIACU).

All the animals were kept inside a solar simulator (designed in the laboratory and fitted with UV lamp) at a distance of 20 cm from the UV light source provided by an array of UVA and UVB (UVitec, Cambridge, UK) lamp with a dose output ratio at 1:4.

The mice were exposed to UV irradiation five days per week starting with 50 mJ/cm^2^ for the first week, with an increase to 55 mJ/cm^2^ at second week, 63 mJ/cm^2^ at third week, 68 mJ/cm^2^ at fourth week, 76 mJ/cm^2^ at fifth week, 81 mJ/cm^2^ at sixth week, and 89 mJ/cm^2^ at seventh to eighth week for total dose of 2850 mJ/cm^2^. The non-irradiated control group was treated identically with the lamp power off. The integrated UV irradiation was measured with a UV meter (Waldmann Medizintechnik, Schwenningen, Germany). The animals were sacrificed three days after the final irradiation to allow the recovery from the acute UV effects. The blood collected via cardiac puncture was allowed to clot, and the serum was obtained by centrifugation at 3000× *g* for 30 min. Aliquots of serum samples were stored at −20 °C until analyzed.

### 4.3. Evaluation of Skin Thickness

Skin thickness was assessed by measuring skin-fold thickness. Briefly, midline dorsal skin of mouse was lifted up at the neck and base of the tail by pinching and skin-fold thickness was measured mid-way between the shoulders and hips using a caliper (Peacock, Ozaki MFG Co. Ltd., Tokyo, Japan). The graders were blinded to the groups of the mice.

### 4.4. Serum Biochemical Analysis

The hepatotoxicity of garlic powder was determined by measuring the activities of alanine transaminase (ALT) and aspartate transaminase (AST) using commercial kits (Sigma-Aldrich, St. Louis, MO, USA).

### 4.5. Morphological and Histopathological Analysis

Morphologic changes of mouse skin surface were observed using a digital microscope (Olympus, Tokyo, Japan).For histopathological assessment, the dorsal skin tissue was fixed in 10% neutral buffered formaldehyde solution, embedded in paraffin and sliced into 5 μm sections. H & E staining was performed to assess epidermal hyperplasia which was measured by epidermal thickness. Verhoeff’s staining was performed using a Verhoeff Van Gieson elastin staining kit (Polysciences Inc., Warrington, PA, USA) to examine elastic fibers.

### 4.6. ROS Production

Skin samples were harvested from the sacrificed mice and the skin tissue (0.4 g) was homogenized (10,000 rpm, 20 s) with Ultra Turrax (T18 Basic, IKA) in nine volumes of cold normal saline (4 °C) to obtain the skin tissue homogenate. The un-dissolved pellet was removed by centrifugation at 3000× *g* for 20 min at 4 °C, and total supernatant was used for the analysis.

A fluorometric assay was used to determine ROS level. Non-fluorescent 2’,7’-dichloro fluorescin diacetate (DCFDA) is oxidized to the highly fluorescent 2′,7′-dichlorofluorescin (DCF) in the presence of esterase and ROS, including lipid peroxides [22]. Briefly, 50 μM DCFDA was added to dorsal skin homogenates to a final volume of 250 μL. Changes in fluorescence intensity were measured every 5 min for 30 min using a fluorescence plate reader (Twinkle LB 970, Berthold Technologies, Bad Wildbad, Germany) at excitation and emission wavelengths of 485 and 530 nm, respectively. Protein concentrations were determined by the Bradford assay.

### 4.7. Lipid Peroxidation and Antioxidant Enzyme Activity

MDA has been identified as the product of lipid peroxidation. MDA levels in the skin and serum were estimated by the thiobarbituric acid assay. To 1 mL of skin tissue homogenate or serum sample, 2 mL TCA-TBA-HCl (15% *w*/*v* TCA and 0.375% *w*/*v* TBA in 0.25 N HCl) reagent was added and heated for 15 min, flocculent precipitate was removed by centrifugation at 1000× *g* for 10 min and absorbance was measured at a 535 nm against a blank containing no tissue homogenate. The MDA concentration was calculated using an extinction coefficient of 1.56 × 10^5^/m·cm.

SOD activity was determined using SOD assay kit (Dojindo Molecular Technologies, Inc., Kumamoto, Japan). Catalase activity was measured according to the method of Aebi [47]. One unit of catalase was defined as the amount of enzyme required to decompose 1 µM of H_2_O_2_ per min. The reaction was started by the addition of 1.0 mL of freshly prepared 20 mM H_2_O_2_. The rate of decomposition of H_2_O_2_ was measured spectrophotometrically at 240 nm for 1 min. The enzyme activities were expressed as U/mg protein.

### 4.8. MMP-1 and MMP-2 Determination

The production of MMP-1 and MMP-2 were determined in skin homogenate supernatant using commercially available ELISA kits (CUSABIO, Inc., Wuhan, Hubei, China).

### 4.9. Quantitative Real Time RT-PCR

Dorsal skin tissues were homogenized with buffer RLT (lysis buffer, Qiagen, Valencia, CA, USA) in ice bath. Total RNA was extracted from dorsal skin tissue lysate using RNeasy^®^ Protect Mini kit (Qiagen, Valencia, CA, USA) to measure the mRNA expression of procollagen I and MMP-1. Complementary DNA was synthesized from mRNA using QuantiTect^®^ Reverse Transcription kit (Qiagen). Real-time PCR was performed using Quanti Tect TM SYBR^®^ Green PCR kit (Qiagen) according to the manufacturer's protocol. The cDNA was amplified for 45 cycles of denaturation (95 °C for 30 s), annealing (58 °C for 30 s), and extension (72 °C for 45 s) using the following primers: GAPDH forward 5′-CAT GGC CTT CCG TGT TCC TA-3′, reverse 5′-GCG GCACGT CAG ATC CA-3′; MMP-1 forward 5′-TTG CCC AGAGAA AAG CTT CAG-3′, reverse 5′-TAG CAG CCC AGA GAAGCA ACA-3′; procollagen type I forward 5′-GGACCT TGG AAG CCT TGG GGA CC-3′, reverse 5′-GGA ATT CGA AACCAC CGG CGT CGA AGG A-3′. Analyses were performed using Rotor-Gene Q^®^ (Qiagen) and gene expression values were calculated based on the comparative ΔΔCT method.

### 4.10. Statistical Analysis

Data were presented as mean ± SD. One-way ANOVA was used to determine treatment effects. Differences among means were inspected using Tukey test and were considered to be significant at *p* < 0.05.

## 5. Conclusions

All together, the findings of this study indicated that dietary supplementation of garlic (1%) is effective in wrinkle improvement by alleviating oxidative stress and dermal extracellular matrix damage, resulting in the improvement of dermal collagen and elastic fiber status in the hairless mice photoaged model. In conclusion, this study suggests a high possibility for the practical use of garlic as an anti-wrinkle agent.
